# 1781. Spatial Epidemiology of Pneumonia Mortality in Korea: Regional Clustering and Bayesian Spatial Regression Analysis for its Determinants

**DOI:** 10.1093/ofid/ofad500.1610

**Published:** 2023-11-27

**Authors:** Trishna Kisiju, Hyemin Song, Soomin Lee, Joonsu Jang, Byung Chul Chun

**Affiliations:** Korea University College of Medicine, Seoul, Seoul-t'ukpyolsi, Republic of Korea; Korea University College of Medicine, Seoul, Seoul-t'ukpyolsi, Republic of Korea; Korea University College of Medicine, Seoul, Seoul-t'ukpyolsi, Republic of Korea; Korea University College of Medicine, Seoul, Seoul-t'ukpyolsi, Republic of Korea; Korea University College of Medicine, Seoul, Seoul-t'ukpyolsi, Republic of Korea

## Abstract

**Background:**

Pneumonia is the third leading cause of mortality in Korea (2021). This can be attributed to the country’s rapid economic growth paired with demographic shift leading to a brisk increase in the aging population. Owing to this, we aimed to examine the regional disparity in pneumonia mortality and the associated determinants

**Methods:**

Municipality-level pneumonia (ICD-10 code, J12-J18) deaths rates (2019) in Korea were extracted from Statistics Korea. Global Moran’s I statistics was used to test for spatial autocorrelation using 999 Monte Carlo simulations. Local Indicators of Spatial Association (LISA) clustering was used to explore the clusters of pneumonia mortality. The determinants were extracted from the Korean Statistical Information Service, including high-risk drinking, smoking rate, regular exercise rate, the prevalence of chronic diseases like hypertension, diabetes, and obesity, the number of doctors per 1000 population, passive smoking from households and workplaces, PM­_10_ level, rate of unmet medical facilities, and the degree of financial independence. Following the selection of potential determinants through non-spatial generalized linear models, a Bayesian spatial regression using the Integrated Nested Laplace Approximation (INLA) was conducted to estimate the regional determinants of pneumonia mortality. The Besag, York, and Mollié (BYM) model was implemented.

**Results:**

A positive spatial autocorrelation was present for pneumonia mortality (I = 0.446, P = 0.001). Regional disparity in pneumonia mortality was evident from LISA clustering, which revealed high-risk clusters in the northeast and central regions of the peninsula (Figure 1). Financial independence was protective (Relative risk (RR) = 0.848, 95% credible interval (CrI) = 0.774–0.928) whereas the prevalence of hypertension was a significant risk factor for pneumonia mortality (RR = 1.116, 95% CrI = 1.029-1.209). Root Mean Square Error (RMSE) in the prediction of pneumonia mortality by the BYM model was minimum (Figure 2-3).

**Figure 1.**
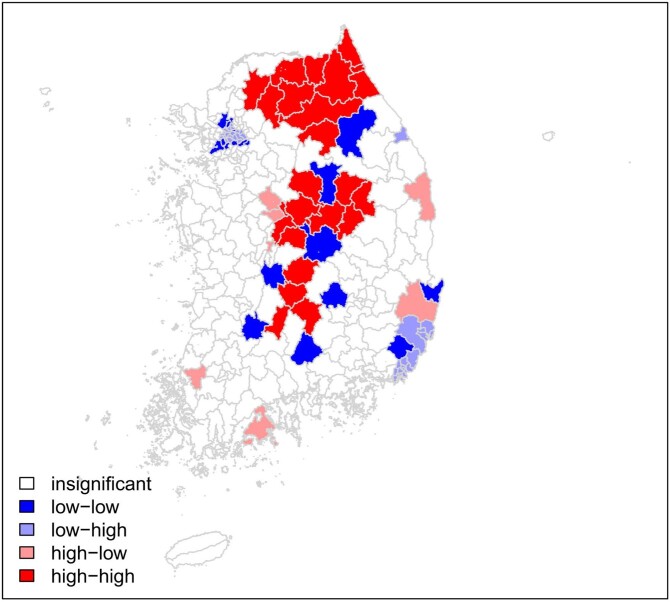
Local Indicators of Spatial Association (LISA) clustering of pneumonia mortality in Korea (2019).

**Figure 2.**
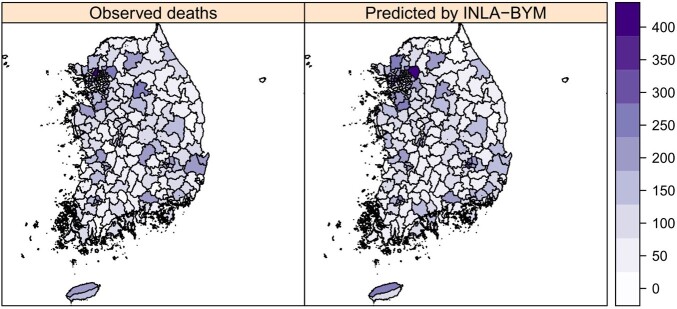
Spatial distribution of the observed pneumonia mortality versus that predicted by INLA-BYM. INLA-BYM, Integrated Nested Laplace Approximation-Besag York Mollié model

**Figure 3.**
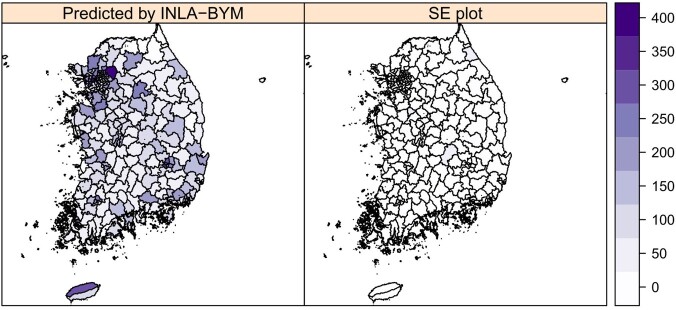
Spatial distribution of the predicted pneumonia mortality versus error in prediction. INLA-BYM, Integrated Nested Laplace Approximation-Besag York Mollié model; SE, Standard error

**Conclusion:**

Regional disparity in pneumonia mortality exists in Korea, which can be explained by the degree of financial independence and the prevalence of hypertension. Policies to curb infection in high-risk areas are encouraged.

**Disclosures:**

**All Authors**: No reported disclosures

